# New Insights into the Population Structure of *Anopheles gambiae s.s.* in the Gulf of Guinea Islands Revealed by *Herves* Transposable Elements

**DOI:** 10.1371/journal.pone.0062964

**Published:** 2013-04-26

**Authors:** Patrícia Salgueiro, Marta Moreno, Frédéric Simard, David O'Brochta, João Pinto

**Affiliations:** 1 Centro de Malária e outras Doenças Tropicais/UEI Parasitologia Médica, Instituto de Higiene e Medicina Tropical, Universidade Nova de Lisboa, Lisboa, Portugal; 2 Centro Nacional de Medicina Tropical, Instituto de Salud Carlos III, Madrid, Spain; 3 MIVEGEC (Maladies Infectieuses et Vecteurs: Ecologie, Genetique, Evolution et Contrôle), UMR IRD224-CNRS5290-UM1-UM2, Institut de Recherche pour le Développement, Montpellier, France; 4 Department of Entomology and The Institute for Bioscience and Biotechnology Research, University of Maryland, College Park, Rockville, Maryland, United States of America; Virginia Tech, United States of America

## Abstract

Transposable elements (TEs) are mobile portions of DNA that are able to replicate and spread in the genome of many organisms. TEs can be used as a means to insert transgenes in insects, being stably inherited throughout generations. *Anopheles gambiae* is the main vector of human malaria in Sub-Saharan Africa. Given the extraordinary burden this disease imposes, the mosquito became a choice target for genetic control approaches with the purpose of reducing malaria transmission. In this study, we investigated the abundance and distribution of *Herves* TE in *An. gambiae s.s.* from Cameroon and four islands in the Gulf of Guinea, in order to determine their genetic structure. We have detected a population subdivision between Equatorial Guinea islands and the islands of São Tomé, Príncipe and mainland. This partitioning associates more with political rather than geographic boundaries, possibly reflecting different mainland source populations colonizing the islands.

## Introduction

Over the past recent years, transgenic technologies have been proposed for malaria vector control [1], [2], [3]. One of the approaches in development consists in altering the vectorial capacity of wild populations of *Anopheles* mosquitoes by delivering and spreading transmission-blocking transgenes (*e.g.* refractoriness to *Plasmodium* infection) [4], [5]. This approach requires gene drive systems and transposable elements (TEs) were one of the first candidate tools to be considered for population replacement [6], [7].

Transposons or TEs are mobile genetic sequences that can replicate and change their relative position within the genome [8]. Most TEs used for insect germline transformation are Class II elements that transpose by a direct DNA ‘cut-and-paste’ mechanism [7], [9], [10], [11]. Within the genome of the main Afrotropical malaria vector *Anopheles gambiae sensu stricto s*everal TEs have been catalogued [12], [13]. Among these, the *Herves* element is a transpositionally active Class II TE isolated from *An. gambiae s.s.* and found in the sibling species *Anopheles arabiensis* and *Anopheles merus* [14]. *Herves* was inferred to be transpositionally active in natural populations of *An. gambiae* based on the high frequency of intact, transposase-encoding *Herves* elements observed and highly polymorphic insertion sites [15].

The dynamics of TEs within populations is determined by the transpositional activity of the element, the strength of self- and host-regulatory mechanisms and the genetic structure of host populations [16]. Sequence and insertion-site polymorphisms have allowed TEs to be used as markers for population genetic studies [17], including in *An. gambiae* [18], [19], [20].

**Figure 1:**
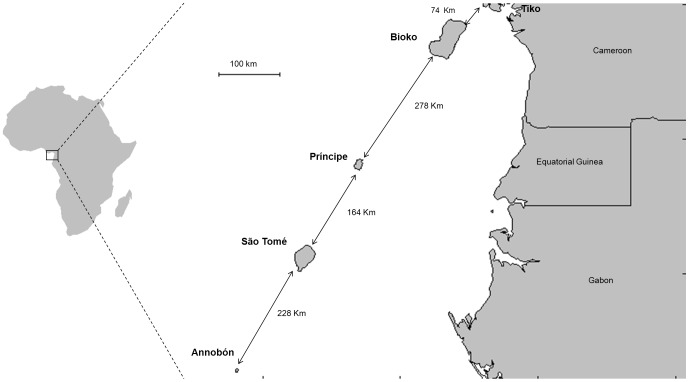
Map of the Gulf of Guinea displaying the islands where collections were conducted and the geographic linear distances among them.

Islands have been considered as candidate sites for experimental releases of transgenic mosquitoes for vector control [21], [22], [23], because the effects of migration from neighboring regions not under control are expected to be minimal. In this context, the islands of the Gulf of Guinea (West Africa) are potential sites that might benefit from malaria control strategies based on transgenic vectors. These islands are volcanic in origin and extend from the coast of Cameroon (Bioko island, 32 km off shore) to 350 km west of the coast of Gabon (Annobón island, Figure 1). Both Bioko and Annobón are part of Equatorial Guinea. Between these two islands, the archipelago of São Tomé and Príncipe is found, which comprises two islands located 140 km apart and about 250 and 225 km, respectively, off the northwestern coast of Gabon. The main malaria vector on these islands is *An. gambiae s.s.* [24], [25]. In the island of Bioko, as well as in mainland (Equatorial Guinea and Cameroon), both M and S molecular forms, considered as incipient species within *An. gambiae s.s.* [26], occur in sympatry [19], [25]. In São Tomé, Príncipe and Annobón only the M form is present [24], [27].

Populations of *An. gambiae s.s.* on Bioko island were found to be genetically differentiated from mainland populations, using microsatellite DNA markers [28]. However, Moreno et al. [27] found a higher degree of genetic isolation of Annobón island when compared with Bioko, which is closest to mainland. Similarly, it has been shown that populations of *An. gambiae s.s.* from the islands of São Tomé and Príncipe are genetically isolated from continental populations [29], [30]. The later authors suggested two main colonization events for *An. gambiae s.s.* into these islands, coincident with episodes of intense human migration [30].

In the islands of the Gulf of Guinea, malaria remains a major cause of morbidity and child mortality. However, in recent years the situation has progressively changed. In São Tomé and Príncipe, a malaria control program has reduced the number of cases by half over the past decade [31]. This program included the use of Indoor Residual Spraying (IRS), Insecticide Treated Nets (ITNs) and artemisinin-combination therapies [32], [33]. Similarly, the prevalence of childhood infection in the island of Bioko has been dropping considerably since the combined implementation of ITNs and IRS [34], [35]. Under these circumstances, the implementation of a transgenic control component might be a promising complement to current malaria control measures, especially when insecticide-based measures are known to be difficult to sustain on the long-term due to issues such as financial constrains or insecticide resistance. However, there are still many issues related with the applicability of transgenic technologies to vector control that require further attention. One of these issues relates with the accurate estimation of the degree of isolation of island populations from mainland [30], [36]. In addition, understanding the dynamics of TE elements in insular populations of *An. gambiae* is critical for developing predictive tools to be used in planning and managing release of transgenic mosquitoes.

In this study, we measured the abundance and site-occupancy frequency distribution of the *Herves* transposon in *An. gambiae s.s.* from Cameroon and the four islands in the Gulf of Guinea, in order to determine the genetic structure of this TE among physically isolated populations and relate with the potential applicability of transposons as gene drive systems for island vector populations.

## Materials and Methods

### Mosquito DNA samples

Mosquito specimens were collected from one continental population of Cameroon, and four island populations of the Gulf of Guinea: Bioko, Príncipe, São Tomé and Annobón (Table 1, Figure 1). Details on the localities and sampling methods have been described elsewhere [27], [29]. Table 1 recalls collection dates and GPS position of collection sites, and Figure 1 displays the geographic linear distances between collection sites.

**Table 1:** Collection data, site occupancy and genetic diversity of the sampled *Anopheles gambiae* populations.

Country	island/ mainland	Locality	Geographical coordinates	Year of colllection	*N*	*k*	dun	hs	95% credibility interval of hs	
									lower	upper
**S. Tomé and Príncipe**	S. Tomé	Riboque	0°19′N/6°43′E	1997	14	15	7.1	0.299	0.238	0.356
				2004	12	15	5.6	0.315	0.241	0.376
	Príncipe	Rua dos Trabalhadores	1°38′N/7°25′E	1998	13	12	4.5	0.302	0.234	0.363
**Equatorial Guinea**	Annobón	Annobón	1°24′S/5°57′E	2004	14	21	7.5	0.291	0.232	0.344
	Bioko	Malabo	3°45′N/8°46′E	2003	14	12	6.0	0.301	0.238	0.358
**Cameroon**	mainland	Tiko	4°05′N/9°21′E	1999	14	15	4.9	0.273	0.210	0.331
					∑ = 81			Hs = 0.297	0.243	0.340

*N*- number of individuals analyzed by TE display per locality; *k* – Number of unique chromosomal sites containing *Herves*; dcn – Diploid copy number of *Herves* [39]; hs – gene diversity with credibility intervals calculated by a Bayesian approach as implemented in HICKORY; Hs – the mean within-population expected heterozygosity ( =  Nei's gene diversity within populations).

No specific permits were required for the described field studies. Mosquito collections were performed in villages either inside houses or outdoors. Informed verbal consent was obtained from household owners to perform the indoor sampling. This type of consent was chosen to avoid potential conflicts due to mistrust in signing official forms by the community. No specific ethics clearance was requested, since all mosquito collections were performed as part of routine mosquito surveillance implemented by the Malaria Control Programs of the regions sampled. The field studies did not involve endangered or protected species.

Extraction of DNA from single mosquitoes was performed by a phenol-chloroform protocol [37]. Species identification and determination of molecular forms were performed by PCR-RFLP according to a previously described protocol [38]. A total of 81 *An. gambiae s.s.* of the M molecular form were analyzed in this study. Sample sizes for each locality are shown in Table 1.

### Transposable element display

PCR-based transposable element display was performed as described by Subramanian et al. [15]. Images of the scanned TE display gels were printed and bands were assigned to a molecular weight based on their mobility relative to the size markers using a standard curve developed for each gel. All samples from each site were analyzed on the same gel, allowing a single standard molecular weight curve to be used to score each dataset. On the basis of the combined results of three TE display experiments, a band was called as present or absent if it was unambiguously present in at least two of the three replicates. Determining the presence of bands in this way resulted in a single scoring matrix that was then used in subsequent analyses.

### Data Analysis

Site occupancy frequency distributions were estimated using the TE display data. To obtain estimates of element frequencies and copy numbers per haploid genome we assumed Hardy–Weinberg equilibrium and followed Wright et al. [39], where the mean number of elements per haploid genome was calculated as described in [40]. In order to discard any locus under selection, we looked for outlier loci using the Dfdist approach with 100.000 simulations [41], [42] in MCHEZA program [43].

Expected heterozygosity *h*s, mean within-population expected heterozygosity *H*s and their 95% credibility values, were calculated using a Bayesian approach with 250.000 generations after a burn-in of 50.000 generations implemented in HICKORY 1.1 [44].

We calculated pairwise Fst estimates as a measure of genetic differentiation based on [45] and tested isolation by distance through the analysis of the correlation coefficient of Fst/(1-Fst) over pairwise geographical distances using Mantel tests [46]. Analysis of Molecular Variance (AMOVA) was used to investigate the genetic structure of the mosquito populations as described in Excoffier et al. [47]. For all these analyses, we used ARLEQUIN 3.1.1 [48]. Permutation-based statistical tests were performed with 50,000 permutations.

For a visual representation of genetic differentiation patterns, we performed a factorial correspondence analysis (FCA) on the multilocus genotype of each individual, using the option that takes into account the population of origin (FCA over populations), as implemented in GENETIX v. 4.05.2 [49].

## Results

### Site occupancy

We detected *Herves* elements in all specimens analyzed by TE display (*N* = 81). A total of 35 sites were detected. Within each sample, element copy number (dcn) ranged from 4.5 to 7.5 per diploid genome (Table 1). The maximum number of elements per individual ranged from six in Príncipe to nine in Annobón.

The TEs with the highest site-occupancy frequency were: *Herves* element with 160 bp (Hv160, showing 6–13 occurrences per sample) and Hv130 (6–14 occurrences per sample) (Table S1, supporting information file). We have found a single element (Hv157) common to all six samples. The sample with the highest number of exclusive sites was Annobón (7, Table 2). The two temporal samples from S. Tomé shared 13 out of 15 possible sites, and these had 10 sites in common with the continental sample (Cameroon, Table 2).

**Table 2:** Distribution of shared *Herves* sites among populations of *An. gambiae* in the Gulf of Guinea.

	Came roon	Bio ko	Prín cipe	S. Tom é 97	S. Tom é04	Anno bón
Cameroon	***2***					
Bioko	5	***1***				
Príncipe	6	4	***3***			
S. Tomé 1997	10	5	8	***1***		
S. Tomé 2004	10	5	9	13	***0***	
Annobón	7	7	7	9	9	***7***

Values in the diagonal represent the number of sites exclusive to the correspondent population.

### Population structure of Herves element

The analysis with Dfdist showed no evidence of sites under selection and all subsequent analyses were thus performed using the 35 detected sites. The estimates of genetic diversity (hs) within each sample ranged from 0.273 in Cameroon to 0.315 in S. Tomé 2004 (Table 1). These values were comparable among samples, judging from the overlapping credibility intervals.

Pairwise Fst-values ranged from −0.080 (Príncipe *vs.* S. Tomé 1997) to 0.574 (Cameroon and S. Tomé 1997 *vs.* Annobón). Comparisons between Annobón and the other samples showed the highest and the only significant Fst estimates (Table 3). The only exception was the comparison of Annobón and Bioko. The two temporal samples from S. Tomé showed no genetic differentiation between them. The Fst values among São Tomé and Príncipe islands (S. Tomé and Príncipe) and the continental Cameroon were negative. The comparison of these samples with Bioko and Annobón showed much higher Fst values. No statistically significant correlation was detected between genetic differentiation and geographic distance (Mantel Test, r = −0.080, *P* = 0.541).

**Table 3:** Estimates of pairwise genetic differentiation among populations of *An. gambiae s.s.* in the Gulf of Guinea, based on *Herves* elements.

	Came roon	Bio ko	Prín cipe	S. To mé 97	S. Tom é04	Ann obón
Cameroon	*-*					
Bioko	0.488	*-*				
Príncipe	−0.080	0.472	*-*			
S. Tomé 1997	−0.077	0.488	−0.080	*-*		
S. Tomé 2004	−0.037	0.324	−0.046	−0.037	*-*	
Annobón	**0.574**	−0.064	**0.559**	**0.574**	**0.418**	*-*

Significant Fst estimates after Bonferroni correction in bold.

**Figure 2:**
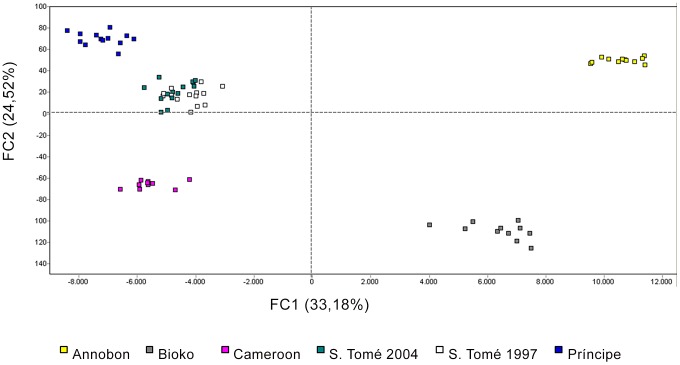
Projection of 81 individual *Herves* genotypes of *Anopheles gambiae* on the principal axes of a factorial component analysis. Each colour corresponds to a sampled island population as indicated in the legend. Inertia percentage values are presented for each factorial component (FC1 and FC2).

Figure 2 shows the first two components of the FCA performed with five clusters corresponding to our samples. The first axis of variation (33.2% on the FC1) visibly splits Annobón and Bioko from the other samples. Also continental Cameroon seems to be closer to São Tomé and Príncipe islands, where the two temporal samples from S. Tomé cluster together. The AMOVA results corroborate the FCA (Table 4). It shows a strong genetic differentiation between Equatorial Guinea islands and the other samples: 56.3% of the total variation was attributed to variation among the two groups and −2.7% of the variance was attributed to population variation within groups (Table 4, group A, *P<*0.001).

**Table 4:** Apportionment of molecular variance measured among populations of *An. Gambiae.*

GroupsTested	Total variance	% amonggroups	*P*	% among populations within groups	% within populations
**A**	0.30	56.3	<0.001	−2.7	46.5
**B**	0.21	−11.8	n.s.	44.5	67.4
**C**	0.26	48.3	<0.05	−3.1	54.8
**D**	0.23	41.8	n.s.	−2.74	60.9

*P* represents the significance of the variation among groups (random > observed). Variation was partitioned in the tested structures.

**A**: group 1: Cameroon, Príncipe, S. Tomé (1997, 2004), group 2: Annobón and Bioko;

**B**: group 1: Cameroon, group 2: all other populations;

**C**: group 1: Cameroon, group 2: S. Tomé and Príncipe, group 3: Annobón and Bioko.

**D**: group 1: Cameroon, group 2: Príncipe, group3: S. Tomé, group 4: Annobón and group 5: Bioko.

## Discussion

We have detected a genetic partitioning between Equatorial Guinea islands (Annobón and Bioko) from the populations of São Tomé and Príncipe and mainland. This was evidenced by both FCA and AMOVA and also by pairwise Fst estimates. Geographic distance was not correlated with genetic differentiation and thus isolation by distance does not seem to influence *Herves* genetic structure. These results only partially agree with previous microsatellite-based studies conducted in the same geographic region. Reimer et al. [28] obtained significant Fst estimates between Bioko island and Cameroon M-form populations ranging from 0.038 to 0.058. These estimates were comparable to Fst estimates (0.023–0.042) obtained between Bioko island and mainland Equatorial Guinea [27]. However, these values were much lower than those recorded between Annobón and mainland (0.187–0.196) or Annobón and Bioko (0.212) [27]. A comparison between populations of São Tomé and Príncipe and Gabon also revealed high Fst values (0.118–0.250) but this study compared M-form island populations with S-form in mainland, which could have inflated differentiation [50]. A subsequent microsatellite analysis based on 13 loci comparing M-form populations from the four Guinean Gulf islands with mainland samples from central and southern Africa also revealed higher differentiation of Annobón (Fst: 0.229–0.271), intermediate for São Tomé and Príncipe (Fst: 0.103–0.255) and lowest in Bioko (0.030–0.080), in agreement with their geographic distance relative to mainland (Pinto et al., unpublished observations).

The patterns of population structure disclosed by the *Herves* TE seem to reflect more political rather than geographic relations among islands. Bioko and Annobón, which belong to the same country, appear more closely related to each other and highly differentiated from mainland or São Tomé and Príncipe, in spite of being the nearest and farthest islands from mainland, respectively. A possible explanation could be a higher passive mosquito dispersal promoted by the movement of Equatorial Guinean nationals between islands. This hypothesis would imply a higher degree of human-mediated dispersal between islands of the same nationality (*i.e.* Bioko-Annobón; São Tomé-Príncipe) rather than between islands in closer geographic proximity (*e.g.* Bioko-Príncipe). However, the differences in mosquito biodiversity between the islands do not support this hypothesis and rather suggest that human-mediated mosquito dispersal between islands is likely to be rare. In São Tomé and Príncipe, the anopheline fauna is represented by only two species: *An. gambiae s.s.* M-form and *Anopheles coustani* [24], [51]. In contrast, at least *Anopheles melas, Anopheles funestus*, *Anopheles smithii* and both M and S forms of *An. gambiae s.s.* have been recorded in Bioko island [28], [52]. These differences are consistent with a general island biogeography model found to occur in the islands of the Guinean Gulf [53], which would otherwise be violated if human-mediated anopheline dispersal was a common event.

Another possibility is that the patterns of population structure reflect different historical mainland source populations from which *Herves* elements have been introduced into São Tomé and Príncipe and Equatorial Guinea islands, respectively. Indeed, both historical and human mtDNA data suggest distinct origins and timings for the arrival of humans in Bioko (*ca.* 10,000 years BP) and São Tomé island (during the 15^th^ century) [54]. Given the synanthropic nature of *An. gambiae s.s.* it is likely that this mosquito also colonized the islands along with the human peopling [29], [30]. Furthermore, sequencing analysis of mtDNA and rDNA markers suggested at least two independent introductions of *An. gambiae s.s.* in São Tomé and Príncipe [30]. The study identified mainland populations from Ivory Coast/Ghana and from Angola as the most likely sources of introduction of this mosquito into São Tomé and Príncipe. While no samples from these countries were included in the present analysis, the low levels of differentiation between São Tomé and Príncipe islands and Cameroon may suggest *An. gambiae* M-form from Cameroon as a source population of *Herves* TEs in São Tomé and Príncipe. These results reinforce the notion of multiple, yet sporadic, colonizations of this vector into these islands. Given these considerations, it is therefore likely that the strong differentiation revealed by *Herves* TE when comparing Bioko and Annobón with São Tomé, Príncipe and Cameroon is the result of different continental origins of these populations. In this context, the analysis of additional mainland samples would be required in order to further clarify the source populations of *An. gambiae s.s.* from Annobón and Bioko.

Finally, we cannot exclude the possibility that differences in the mode of evolution and inheritance may also contribute for the apparent discrepancy between patterns of population structure obtained by the *Herves* TE and by previous microsatellite based analyses. However, little is known about the mechanisms of regulation of *Herves* TE activity, which poses difficulties for comparing these markers.

The low differentiation observed between the two temporal samples from S. Tomé provides no evidence for significant demographic changes in the mosquito population between 1997 and 2004 or for high rates of *Herves* transposition during this period. This result is consistent with the large estimates of current effective population size obtained for *An. gambiae* M-form in São Tomé island [29], [50], comparable to those observed in mainland for the members of *An. gambiae* complex [55], [56]. The relative demographic stability of *An. gambiae* suggested by this and previous studies in São Tomé and Príncipe is based on the analysis of samples for which collections pre-date the recent implementation of vector control measures that resulted in a significant malaria decrease on the islands [32], [33]. In this context, it would be interesting to analyze the genetic variation of *Herves* TE in samples collected post-intervention as a means to assess the usefulness of TE in determining the impact of vector control in the genetic structure of *An. gambiae.*

In this study, locally fixed *Herves*-occupied sites were rare and sites occupied in only a few individuals were very common in the sampled populations. This suggests that *Herves* remains active within these island mosquito populations, as was also observed in previous analyses of other mainland African *An. gambiae* populations [15], [40]. Following WHO's recommendations [22], transgenic mosquitoes use should involve several phases of field release under conditions that limit spread into the environment. In this context, remote islands are considered as suitable candidate sites. Accordingly, the most recent open-field trials to attempt transgenic-based vector control involved island mosquito populations [23]. In agreement with previous studies based on other genetic markers [27], the analysis of *Herves* TE suggests Annobón as probably the most adequate of the four islands of the Gulf of Guinea for experimental release of transgenic vectors: it is the most remote island with *An. gambiae* displaying the highest degree of genetic isolation and the highest amount of *Herves* insertion site polymorphism. However, the patterns of population structure on the islands of the Guinean Gulf disclosed in this study by *Herves* TE are more likely to reflect different origins of mosquito colonization rather than levels of contemporary gene flow. These results should therefore be interpreted with caution. Further analyses involving comparisons with more mainland samples and combining different genetic markers are required to clarify the degree of isolation of these islands.

## Supporting Information

Table S1 **Distribution of Herves sites detected over the six sampled populations.**
(DOCX)
