# Supplementary material for: New Insights into the Population Structure of Anopheles gambiae s.s. in the Gulf of Guinea Islands Revealed by Herves Transposable Elements
Source: PLoS One. 2013 Apr 26;8(4):e62964. doi: 10.1371/journal.pone.0062964 (PMC3637158; doi:10.1371/journal.pone.0062964)
Supplement: Table S1 — Distribution of Herves sites detected over the six sampled populations. (DOCX) [file pone.0062964.s001.docx]

# Supporting Information file

**Table S1. Distribution of *Herves* sites detected over the six sampled populations**

| **Populations** | ***Herves* sites detected in base pairs** | | | | | | | | | | | | | | | | | | | | | | | | | | | | | | | | | | | |
| --- | --- | --- | --- | --- | --- | --- | --- | --- | --- | --- | --- | --- | --- | --- | --- | --- | --- | --- | --- | --- | --- | --- | --- | --- | --- | --- | --- | --- | --- | --- | --- | --- | --- | --- | --- | --- |
|  | **1**  **1**  **3** | **1**  **1**  **5** | **1**  **1**  **8** | **1**  **2**  **0** | **1**  **2**  **3** | **1**  **2**  **5** | **1**  **3**  **0** | **1**  **3**  **8** | **1**  **4**  **0** | **1**  **4**  **5** | **1**  **4**  **8** | **1**  **5**  **0** | **1**  **5**  **3** | **1**  **5**  **5** | **1**  **5**  **7** | **1**  **6**  **0** | **1**  **6**  **2** | **1**  **6**  **5** | **1**  **7**  **5** | **1**  **8**  **0** | **1**  **8**  **5** | **1**  **9**  **5** | **2**  **0**  **0** | **2**  **0**  **5** | **2**  **1**  **0** | **2**  **1**  **5** | **2**  **2**  **0** | **2**  **2**  **5** | **2**  **3**  **0** | **2**  **5**  **0** | **2**  **6**  **0** | **2**  **6**  **5** | **2**  **7**  **0** | **2**  **7**  **5** | **2**  **8**  **5** | **Total** |
| Annobón | 1 | 1 | 9 | 1 | 1 | 9 | 9 | 3 | 1 | 1 |  |  | 3 |  | 10 | 13 | 1 |  |  |  | 3 |  |  | 2 | 2 |  |  |  |  | 2 |  | 2 | 9 | 1 |  | 21 |
| Príncipe |  |  | 4 | 3 |  |  | 11 |  | 4 |  |  | 1 |  |  | 1 |  |  |  |  | 3 | 5 |  |  |  |  |  |  | 1 | 5 |  | 2 |  |  | 9 |  | 12 |
| S. Tomé 1997 |  | 2 | 4 | 2 |  |  |  | 6 | 1 |  |  |  |  | 5 | 1 | 12 |  |  |  | 7 | 1 |  |  |  |  | 1 | 10 | 10 |  |  |  |  |  | 13 | 2 | 15 |
| S. Tomé 2004 |  |  | 6 | 1 |  |  | 6 | 3 | 2 |  |  |  |  | 3 | 2 | 6 |  |  |  | 4 | 6 |  |  |  |  | 3 | 4 | 5 |  |  |  |  |  | 7 |  | 14 |
| Bioko |  | 6 |  | 2 |  | 2 | 14 |  |  | 1 |  |  |  | 6 | 9 |  |  | 1 | 1 | 12 |  | 3 |  |  | 3 |  |  |  |  |  |  |  |  |  |  | 12 |
| Cameroon |  |  | 1 |  |  |  |  |  | 7 |  | 1 |  |  | 14 | 1 | 11 | 1 | 1 |  | 1 | 4 | 1 | 1 |  |  | 2 | 1 |  |  |  |  |  |  | 2 |  | 15 |
| Σ | 1 | 9 | 24 | 9 | 1 | 11 | 40 | 12 | 15 | 2 | 1 | 1 | 3 | 28 | 24 | 42 | 2 | 2 | 1 | 27 | 19 | 4 | 1 | 2 | 5 | 6 | 15 | 16 | 5 | 2 | 2 | 2 | 9 | 32 | 2 |  |
| Pop | 1 | 3 | 5 | 5 | 1 | 2 | 4 | 3 | 5 | 2 | 1 | 1 | 1 | 4 | 6 | 4 | 2 | 2 | 1 | 5 | 5 | 2 | 1 | 1 | 2 | 3 | 3 | 3 | 1 | 1 | 1 | 1 | 1 | 5 | 1 |  |
| Σ: sum of occurrences of each *Herves* site over all populations; Pop: number of populations where each *Herves* site was detected; Total: total number of sites detected in each population | | | | | | | | | | | | | | | | | | | | | | | | | | | | | | | | | | | |  |
